# Ecotoxicological effects of heavy metal contamination on reproductive health and gamete quality in female Nile tilapia (*Oreochromis niloticus*) from the Brantas River Basin, Indonesia

**DOI:** 10.14202/vetworld.2025.1634-1643

**Published:** 2025-06-19

**Authors:** Habib Syaiful Arif Tuska, Gretania Residiwati, Anik Martinah Hariati, Anwar Sanusi, Gatot Ciptadi, Barlah Rumhayati, Hendra Susanto, Aulanni’am Aulanni’am

**Affiliations:** 1 Doctoral Program in Environmental Science, Universitas Brawijaya, Malang, Indonesia; 2 Department of Veterinary Reproduction, Faculty of Veterinary Medicine, Universitas Brawijaya, Malang, Indonesia; 3 Department of Anatomy, Histology, and Embryology, Faculty of Veterinary Medicine, Universitas Brawijaya, Malang, Indonesia; 4 Department of Aquaculture Faculty of Fisheries and Marine Science, Universitas Brawijaya, Malang, Indonesia; 5 Manpower Planning and Development Agency, Ministry of Manpower of the Republic of Indonesia, Jakarta, Indonesia; 6 Department of Government Politics, Faculty of Social and Political Sciences, Universitas Brawijaya, Malang, Indonesia; 7 Department of Animal Reproduction and Breeding, Faculty of Animal Science, Universitas Brawijaya, Malang, Indonesia; 8 Department of Chemistry, Faculty of Mathematics and Natural Sciences, Universitas Brawijaya, Malang, Indonesia; 9 Department of Biology, Faculty of Mathematics and Natural Sciences, Universitas Negeri Malang, Malang, Indonesia

**Keywords:** Brantas River, cadmium, heavy metal contamination, histopathology, iron, Nile tilapia, oocyte quality, reproductive toxicity

## Abstract

**Background and Aim:**

Heavy metal pollution in freshwater ecosystems poses a serious threat to aquatic biodiversity and food safety. This study assessed the bioaccumulation of lead, cadmium (Cd), iron (Fe), and copper, as well as their effects on histopathological alterations in vital organs and gamete quality, in female Nile tilapia (*Oreochromis niloticus*) from the Brantas River Basin, East Java, Indonesia.

**Materials and Methods:**

Water and fish samples were collected from five sites with varying levels of industrial and residential activity. Heavy metal concentrations were analyzed through atomic absorption spectrophotometry. Histopathological evaluations were performed on gills, liver, and ovaries, and gamete quality was assessed based on oocyte diameter, germinal vesicle breakdown (GVBD), survival, and abnormality rate. Statistical analyses included a one-way analysis of variance, Kruskal-Wallis tests, and Spearman correlation.

**Results:**

The highest concentrations of Cd and Fe were found in Kalisari, corresponding with pronounced histopathological lesions in fish, including necrosis, inflammation, and hyperplasia in vital organs. Oocytes from heavily polluted sites exhibited significantly reduced diameters, GVBD rates, and survival rates, alongside increased abnormalities. Cd and Fe levels exhibited strong positive correlations with organ damage and negative correlations with gamete quality, particularly oocyte survival (Fe: r = −0.900).

**Conclusion:**

Exposure to elevated levels of Cd and Fe significantly impairs the physiological and reproductive health of female Nile tilapia in the Brantas River. The observed tissue damage and reproductive disruption underscore the ecological and public health risks associated with unchecked industrial discharge. Long-term biomonitoring and targeted pollution control strategies are urgently required to safeguard aquatic life and reduce health risks under the One Health framework.

## INTRODUCTION

Heavy metal contamination in freshwater ecosystems represents a critical global environmental challenge, with profound implications for aquatic biodiversity and human health [1–5]. Toxic elements such as lead (Pb), cadmium (Cd), iron (Fe), and copper (Cu) can cause severe ecological damage when they enter aquatic environments. These contaminants are predominantly introduced through anthropogenic activities, including industrial effluents, agricultural runoff, municipal wastewater, and mining operations [6, 7]. Once released into freshwater systems, heavy metals tend to bioaccumulate in aquatic organisms, particularly fish, where they disrupt physiological processes and compromise organismal health, thereby posing risks to human populations that depend on fish as a primary source of protein [8].

Freshwater ecosystems are particularly vulnerable to heavy metal pollution, a condition that is further exacerbated by heavy rainfall events, which enhance the mobilization and dispersion of contaminants into rivers and lakes. In aquatic organisms, metals such as Pb, Cd, Fe, and Cu act as xenobiotics, accumulating in critical organs, including the gills, liver, kidneys, and gonads, and inducing oxidative stress, cellular damage, and inflammatory responses. Accumulation in reproductive tissues is especially concerning, as it can impair gametogenesis, compromise gamete quality, and reduce reproductive success, with potential long-term consequences for fish populations and food security. In addition, the biomagnification of heavy metals through trophic levels poses significant public health risks to consumers of contaminated fish products [9, 10].

Numerous studies have documented the widespread occurrence and deleterious effects of heavy metal pollution on fish health in both developed and developing regions [11, 12]. In Southeast Asia, particularly in Indonesia, this issue has escalated into a serious environmental and public health concern. For instance, the Brantas River in East Java has been identified as a major hotspot for heavy metal contamination, with documented adverse impacts on local fish populations and the communities that rely on these aquatic resources for their livelihood and nutrition [13, 14].

Despite extensive documentation of heavy metal contamination in freshwater environments globally, relatively few studies have explored the specific reproductive toxicity and histopathological consequences of such contamination in female *Oreochromis niloticus* within highly industrialized river basins in Southeast Asia. Most existing literature focuses on either water quality assessment or generalized fish health parameters, with limited emphasis on organ-specific pathology and reproductive impairments under field conditions. Moreover, current research rarely integrates multi-organ histopathological analysis with detailed gamete quality metrics, thereby limiting the understanding of sub-lethal but ecologically significant impacts of heavy metals. In the context of Indonesia, the Brantas River, one of the country’s most economically and ecologically important freshwater systems has been increasingly subjected to anthropogenic pollution. However, empirical data on how chronic exposure to metals such as Cd and Fe influences the reproductive physiology of Nile tilapia, a key aquaculture species in the region, remain scarce.

This study aims to investigate the ecotoxicological effects of heavy metal contamination, specifically Pb, Cd, Fe, and Cu, on the reproductive health and gamete quality of female Nile tilapia *(O. niloticus*) in the Brantas River Basin, Indonesia. By combining heavy metal concentration profiling with histopathological evaluations of the gills, liver, and ovaries, and quantitative assessments of oocyte characteristics (diameter, germinal vesicle breakdown [GVBD], viability, and morphological abnormality), this research seeks to elucidate the relationship between environmental contamination and reproductive dysfunction. The findings aim to contribute to a more comprehensive understanding of sub-lethal toxic effects on freshwater fish reproduction, inform ecological risk assessments, and support the development of targeted conservation and remediation strategies within the One Health framework.

## MATERIALS AND METHODS

### Ethical approval

All experimental procedures involving live animals were reviewed and approved by the Institutional Animal Care and Use Committee of Universitas Brawijaya, Malang, Indonesia (Approval No. 201-KEP-UB-2024), in accordance with national animal welfare regulations.

### Study period and location

The study was conducted from February to September 2024 in the Brantas River Basin, East Java, Indonesia, to encompass seasonal variability in rainfall and runoff. Sampling was performed bimonthly at five ecologically distinct sites that represent a gradient of anthropogenic influences, ranging from the relatively unpolluted upstream source at Sumber Brantas (Bumiaji District, Batu) to downstream areas impacted by industrial, agricultural, and residential activities. The sites included Selorejo (Pandansari Village, Ngantang District), Kalisari (Mangliawas Village, Pakistan District), Sengguruh (Sengguruh Village, Kepanjen District), and Karangkates (Karangkates Village, Sumberpucung District). Nile tilapia (*O. niloticus*), a key aquaculture species in the region, served as the bioindicator organism, providing insights into the spatial distribution of heavy metals (Pb, Cd, Fe, and Cu) and their effects on fish health.

### Study design and sampling sites

This study investigated the bioaccumulation of heavy metals (Pb, Cd, Fe, and Cu) and their impact on fish health across five ecologically distinct sites within the Brantas River Basin, East Java, Indonesia. Sampling locations were strategically selected based on gradients of anthropogenic activity, including industrial, agricultural, and residential influences.

The five sites included:

1. Control - Sumber Brantas, the river’s origin (Bumiaji District, Batu; −7.754075, 112.526640)
2. Selorejo - Pandansari Village, Ngantang District, Malang (−7.873486, 112.356285)
3. Kalisari - Mangliawan Village, Pakis District, Malang (−7.950162, 112.664542)
4. Sengguruh - Sengguruh Village, Kepanjen District, Malang (−8.182920, 112.549957)
5. Karangkates - Karangkates Village, Sumberpucung District, Malang (−8.156390, 112.434519).

The control site (Sumber Brantas) represented the least polluted upstream source, while the other sites were chosen based on their proximity to known pollution sources. Nile tilapia (*O. niloticus*), a species of high aquacultural relevance in the region, was used as the bioindicator organism. Sampling was conducted bimonthly from February to September 2024 to account for seasonal variations in pollutant exposure associated with rainfall and runoff.

### Water sampling and heavy metal analysis

At each site, five liters of surface water were collected in acid-washed polyethylene containers, sealed with aluminum foil to prevent contamination, and kept on ice during transport to the Integrated Service Laboratory, Faculty of Agricultural Technology, Universitas Brawijaya. Upon arrival, samples were acidified to pH <2 using ultrapure nitric acid (Merck, Germany) and stored at 4°C until further analysis. The concentrations of Pb, Cd, Fe, and Cu were quantified using flame atomic absorption spectrophotometry (AAS) (AAS; Shimadzu AA–7,000), in accordance with EPA Method 200.7 (United States Environmental Protection Agency, USA).

### Fish collection and handling

Twenty-five reproductively mature female *O. niloticus* (five per site) were captured using 0.2 cm mesh gill nets. Only individuals within the length range of 15–20 cm and the weight range of 100–150 g were selected to ensure uniformity in reproductive status. Fish were transported live in oxygenated polyethylene bags within insulated Styrofoam containers to minimize stress and preserve physiological integrity. Upon arrival at the laboratory, fish were anesthetized and euthanized using tricaine methanesulfonate (MS) (MS-222, 100 mg/L; Phy Edumedia, Indonesia), following the protocol described by Carter *et al*. [15].

### Histopathological examination

To assess tissue-level impacts of heavy metal exposure, the gills, liver, and ovaries were aseptically excised and fixed in 10% neutral-buffered formalin for 24 h at ambient temperature. Tissues underwent standard histological processing: dehydration through a graded ethanol series (70% to absolute), clearing in xylene (three changes, 20 min each), and embedding in paraffin wax at 60°C across three successive stages (each 1 h). Subsequently, tissues were sectioned into 5 μm thick slices using a rotary microtome, mounted on poly-L-lysine-coated slides, and stained with hematoxylin and eosin.

Microscopic evaluation was performed at 100× and 400× magnification across at least five non-overlapping fields per section. Histopathological alterations for gills, liver, and ovaries, including inflammation, necrosis, hyperemia, and hyperplasia, were semi-quantitatively scored on a scale from 0 (normal) to 3 (severe), as adapted from Gibson-Corley *et al*. [16] (Tables 1–3). Scoring was conducted independently by two patholo-gists who were blinded to the sampling location.

**Table 1:** Histopathological scoring of the gills [16].

Score	Severity	Level of changes			

		Inflammation (%)	Necrosis (%)	Hyperemia (%)	Hyperplasia (%)
0	Normal	0–1	0–1	0–1	0–1
1	Mild	2–15	2–20	2–20	2–40
2	Moderate	16–25	21–30	21–30	41–60
3	Severe	>25	>30	>30	>60

**Table 2:** Histopathological scoring of the liver [16].

Score	Severity	Level of changes		

		Inflammation (%)	Necrosis (%)	Hyperemia
0	Normal	0–1	0–1	0–1
1	Mild	2–40	2–40	2–40
2	Moderate	41–60	41–60	41–60
3	Severe	>60	>60	>60

**Table 3:** Histopathological scoring of ovaries [16].

Score	Severity	Level of changes			

		Inflammation (%)	Necrosis (%)	Hyperemia (%)	Hyperplasia (%)
0	Normal	0–1	0–1	0–1	0–1
1	Mild	2–15	2–20	2–20	2–40
2	Moderate	16–25	21–30	21–30	41–60
3	Severe	>25	>30	>30	>60

### Gamete quality assessment

A total of 450 oocytes in phases III-IV (18 per fish) were extracted from ovarian tissue, corresponding to the vitellogenic and pre-maturation stages, which are known for their high metabolic activity and environmental sensitivity [17–19]. Dissection was performed under a stereomicroscope, and oocytes were gently rinsed in phosphate-buffered saline (PBS) (PBS; pH 7.4) to remove residual tissue.

Gamete quality was evaluated using four key parameters: Oocyte diameter (OD), GVBD rate, survival rate, and morphological abnormalities. OD was measured at 40× magnification using Optilab Viewer software and calibrated against a micrometer scale in ImageJ (Wayne Rasband, USA). The average of two perpendicular diameters was recorded using the method described by Pedersen *et al*. [20].

GVBD, an indicator of oocyte maturation to phase V, was visually assessed under a microscope at 40× magnification. Mature oocytes were identified by their transparent, yellowish appearance, and the absence of a visible germinal vesicle. GVBD rate was calculated as the percentage of oocytes undergoing breakdown:

GVBD (%) = (Number of GVBD oocytes/Total oocytes) × 100% [21].

Viability was determined using the Trypan Blue exclusion method (0.1%, Phy Edumedia, Indonesia), where unstained oocytes were considered viable and blue-stained oocytes non-viable. The survival rate was expressed as the proportion of viable oocytes relative to the total count [22].

Oocyte morphology was examined at 100× magnification and categorized as normal or abnormal. Abnormalities included cytoplasmic vacuolation, membrane rupture, and the presence of inclusion bodies, following the criteria established by Lasienė *et al*. [23].

### Statistical analysis

All data were analyzed using IBM SPSS Statistics v23.0 (IBM Corp., Armonk, NY, USA). Data normality was assessed using the Shapiro-Wilk test (p > 0.05). Parametric variables were analyzed using a one-way analysis of variance followed by least significant difference *post hoc* test (p < 0.05). For non-normally distributed data, the Kruskal-Wallis test was employed, followed by Mann-Whitney U pairwise comparisons (p < 0.05).

Effect sizes and 95% confidence intervals were calculated to estimate the strength of differences between groups. In addition, Spearman’s correlation analysis (p < 0.05) was conducted to evaluate the relationships between heavy metal concentrations and biological parameters, including histopathological and gamete quality indices.

## RESULTS

### Heavy metal concentrations in water

**Figure 1:**
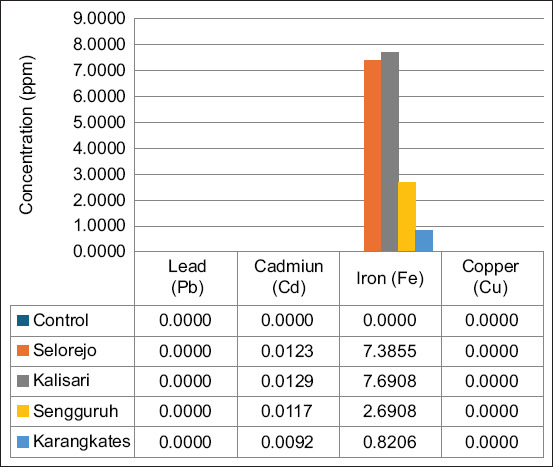
The different concentrations (ppm) of heavy metals (lead, cadmium, iron, copper) from five (5) different sites along the Brantas River.

Figure 1 illustrates the concentrations of Pb, Cd, Fe, and Cu in water samples collected from five locations along the Brantas River. The results showed distinct site-specific variations in heavy metal concentrations. Pb and Cu were either negligible or undetectable across all sites, with values consistently recorded at 0.0000 ppm. The highest Cd concentration was observed at Kalisari (0.0129 ppm), followed by Selorejo (0.0123 ppm), Sengguruh (0.0117 ppm), and Karangkates (0.0092 ppm). In contrast, Fe concentrations were substantially higher at Kalisari (7.6908 ppm) and Selorejo (7.3855 ppm), while significantly lower levels were recorded at Sengguruh (2.6908 ppm) and Karangkates (0.8206 ppm).

### Gill histopathology in female *O. niloticus*

**Figure 2:**
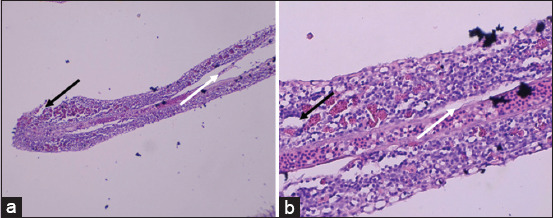
Histopathology of the gill on female Nile tilapia: (a) 100× magnification and (b) 400× magnification; secondary lamellar hyperplasia (white arrow); secondary lamellar necrosis (black arrow) (Personal Documentation, 2024).

**Figure 3:**
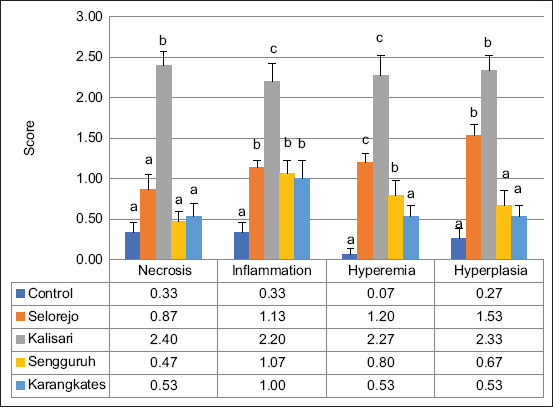
Graphical result of gill histopathology in female Nile tilapia; different subscripts indicate significant differences (p < 0.05) for each observed parameter among the locations.

Histopathological examination of gill tissues revealed significant alterations, including necrosis, inflammation, hyperemia, and hyperplasia (Figure 2). These pathological features were further quantified and are presented in Figure 3.

Nile tilapia from Kalisari exhibited the most severe gill damage, with significantly higher necrosis scores (2.40 ± 0.163) compared to Selorejo (0.87 ± 0.192), Sengguruh (0.47 ± 0.133), Karangkates (0.53 ± 0.165), and the control group (0.33 ± 0.126). Inflammation scores were also highest in Kalisari (2.20 ± 0.223). No significant differences in inflammation were found among Selorejo (1.13 ± 0.091), Sengguruh (1.07 ± 0.153), and Karangkates (1.00 ± 0.220). Hyperemia and hyperplasia scores were elevated in Kalisari (2.27 ± 0.248 and 2.53 ± 0.187, respectively) and moderately high in Selorejo (1.20 ± 0.107 and 1.53 ± 0.133). Control and Karangkates exhibited similar, significantly lower values (hyperemia: 0.07 ± 0.07 and 0.27 ± 0.13; hyperplasia: 0.27 ± 0.12 and 0.53 ± 0.13, respectively).

### Liver histopathology in female *O. niloticus*

**Figure 4:**
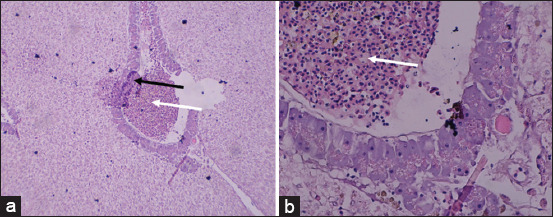
Histopathology of the liver of female Nile tilapia: (a) 100× magnification and (b) 400× magnification; periportal inflammation (white arrow); central vein hyperemia (black arrow) (Personal Documentation, 2024).

**Figure 5:**
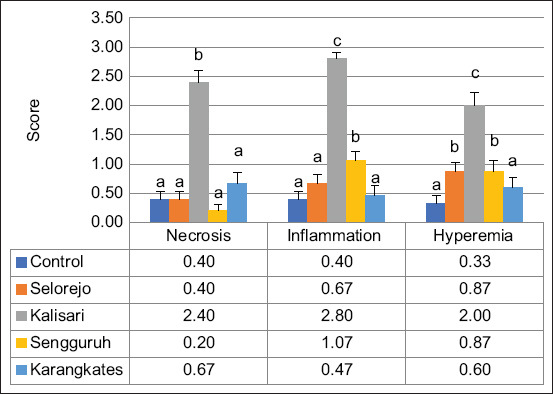
Graphical result of liver histopathology in female Nile tilapia; different subscripts indicate significant differences (p < 0.05) for each observed parameter among the locations.

The liver tissues of Nile tilapia from polluted sites exhibited clear pathological changes, particularly periportal inflammation and central vein hyperemia (Figure 4), with detailed scores illustrated in Figure 5.

Fish from Kalisari recorded the most severe liver pathology, with necrosis (2.40 ± 0.19), inflammation (2.80 ± 0.107), and hyperemia (2.00 ± 0.218) scores significantly higher than those from other locations. In contrast, liver tissues from Karangkates showed no significant difference from the control group in any of the histopathological parameters: Necrosis (0.67 ± 0.187 and 0.40 ± 0.131), inflammation (0.47 ± 0.165 and 0.40 ± 0.131), and hyperemia (0.60 ± 0.163 and 0.33 ± 0.126).

### Ovarian histopathology in female *O. niloticus*

**Figure 6:**
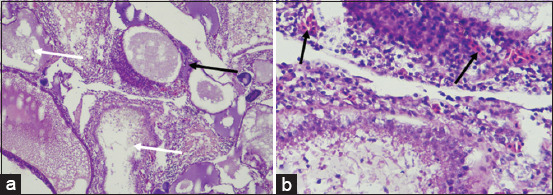
Histopathology of the ovaries in female Nile tilapia: (a) 100× magnification and (b) 400× magnification; atretic follicles (white arrow); hyperemia (black arrow) (Personal Documentation, 2024).

**Figure 7:**
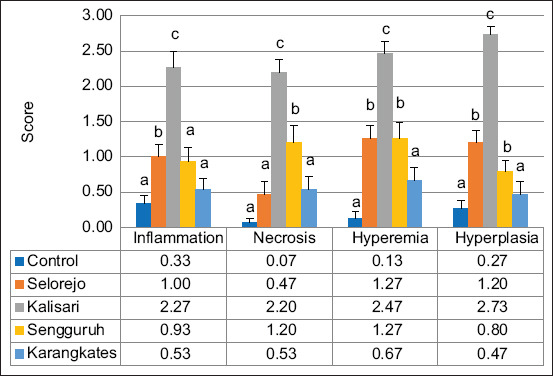
Graphical result of ovary histopathology in Nile tilapia; different subscripts indicate significant differences (p < 0.05) for each observed parameter among the locations.

Ovarian tissues of Nile tilapia exposed to heavy metals displayed multiple pathological lesions, including atretic follicles and extensive hyperemia (Figure 6), with quantitative scores presented in Figure 7.

The highest severity of ovarian damage was observed in Kalisari, with inflammation (2.27 ± 0.288), necrosis (2.20 ± 0.175), hyperemia (2.47 ± 0.165), and hyperplasia (2.73 ± 0.118) all significantly elevated. Inflammation scores in Sengguruh (0.93 ± 0.206) and Karangkates (0.53 ± 0.165) were not significantly different from the control group (0.33 ± 0.126). Moreover, ovarian quality in Karangkates closely resembled that of the control group for necrosis (0.53 ± 0.192 vs. 0.07 ± 0.167), hyperemia (0.67 ± 0.187 vs. 0.13 ± 0.091), and hyperplasia (0.47 ± 0.192 vs. 0.27 ± 0.118).

### Effects of heavy metals on oocyte quality

**Figure 8:**
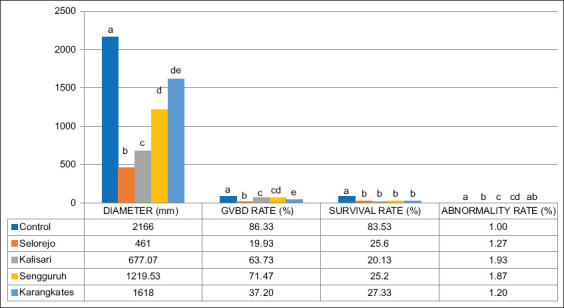
Graphical results of oocyte diameter, germinal vesicle breakdown-, survival-, and abnormality rates in Nile tilapia, different subscripts indicate significant differences (p < 0.05) for each observed parameter among the locations.

Gamete quality assessment, based on OD, GVBD rate, survival rate, and abnormality rate, showed significant variability across sites (Figure 8).

Oocytes from the control group had the largest mean diameter (2166 ± 7.719 μm). Karangkates (1618 ± 19.325 μm) and Sengguruh (1219.53 ± 156.171 μm) showed reduced diameters, with no significant difference between them. The highest GVBD rate was observed in the Control group (86.33% ± 0.82%). Kalisari (63.73% ± 2.912%) and Sengguruh (71.47% ± 4.14%) did not differ significantly in GVBD values. Survival rates were also highest in the Control group (85.33% ± 0.48%). Among other sites, no significant difference was found between Selorejo (25.6% ± 3.28%), Kalisari (20.13% ± 1.68%), Sengguruh (25.2% ± 2.82%), and Karangkates (27.33% ± 2.31%). Oocyte abnormality rates were lowest in the Control group (1.00% ± 0.00%), with no significant difference from Karangkates (1.20% ± 0.11%).

### Correlation between heavy metals and biological parameters

Table 4 summarizes the Spearman correlation analysis between Cd, Fe, and various histological and reproductive parameters in female Nile tilapia.

**Table 4:** Correlation analysis of cadmium and Iron content with multiple parameters in female Nile tilapia.

CO	GN	GI	GHM	GHP	HN	HI	HHM	OI	ON	OHM	OHP	OD	OG	OSR	OAR
Cd	0.769	0.787	0.895*	0.945*	0.564	0.668	0.789	0.819	0.614	0.85	0.849	−0.766	−0.453	−0.629	0.528
Fe	0.900*	1**	1**	1**	0.359	0.900*	0.975**	1**	0.7	0.975**	1**	−0.7	−0.5	−0.900*	0.900*

CO=Correlation, Cd=Cadmium, Fe=Iron, GN=Gill necrosis, GI=Gill inflammation, GHM=Gill hyperemia, GHP=Gill hyperplasia, HN=Liver necrosis, HI=Liver inflammation, HHM=Liver hyperemia, OI=Ovary inflammation, ON=Ovary necrosis, OHM=Ovary hyperemia, OHP=Ovary hyperplasia, OD=Oocyte diameter, OG=Oocyte GVBD rate, OSR=Oocyte survival rate, OAR=Oocyte abnormality rate,

* Correlation is significant at the 0.05 level,

** Correlation is significant at the 0.01 level, −: An increase in the row variable is followed by a decrease in the column variable. GVBD=Germinal vesicle breakdown

Cd showed strong positive correlations with gill hyperemia (r = 0.895) and hyperplasia (r = 0.945), indicating that increasing Cd levels were associated with greater histopathological damage. Fe exhibited even broader correlations, including gill necrosis (r = 0.900), inflammation (r = 1.000), hyperemia (r = 1.000), and hyperplasia (r = 1.000). Similarly, strong correlations were found between Fe concentrations and liver inflammation (r = 0.900) and hyperemia (r = 0.975). Ovarian inflammation (r = 1.000), hyperemia (r = 0.975), and hyperplasia (r = 1.000) were also strongly linked to Fe levels.

Both Cd and Fe demonstrated significant negative correlations with gamete quality metrics. Increased concentrations of these metals were associated with reduced OD, GVBD rate, and survival. Notably, Fe exhibited a particularly strong negative correlation with oocyte survival (r = −0.900), indicating a clear decline in reproductive viability with elevated exposure.

## DISCUSSION

This study aimed to assess the ecotoxicological impact of heavy metal contamination, specifically Pb, Cd, Fe, and Cu, on the reproductive health and gamete quality of female Nile tilapia (*O. niloticus*) inhabiting the Brantas River Basin in East Java, Indonesia.

### Spatial variation in heavy metal contamination

The findings reveal significant spatial heterogeneity in heavy metal concentrations across the five sampling sites, with Fe being the most prevalent contaminant. In contrast, Pb and Cu were consistently recorded at negligible or undetectable levels. Kalisari exhibited the highest concentrations of Cd and Fe, likely attributable to its proximity to industrial and residential zones. This observation aligns with a previous report by Prayoga [24], which identified anthropogenic discharge as a principal contributor to riverine pollution in Indonesia.

### Histopathological alterations in target organs

Nile tilapia from contaminated sites, particularly Kalisari, exhibited severe histopathological changes in the gills, liver, and ovaries. Gills, being the primary site of metal uptake, demonstrated necrosis, inflammation, hyperemia, and hyperplasia [25–27]. The liver, a central organ for detoxification, showed pronounced necrosis and inflammation, consistent with established literature on the hepatotoxic effects of metal exposure [28–30]. Ovarian tissue exhibited inflammation and atresia, supporting earlier findings that link heavy metal exposure with gonadal dysfunction and impaired oogenesis [1, 31].

### Mechanisms of toxicity

Heavy metal toxicity in fish is primarily mediated by oxidative stress. The generation of reactive oxygen species disrupts cellular homeostasis, leading to lipid peroxidation, protein degradation, and DNA damage [32]. Although fish may activate antioxidant enzymes such as superoxide dismutase and catalase as a compensatory response, this defense is often insufficient against sustained exposure. Concurrently, inflammation, leukocyte infiltration, and cellular degeneration further aggravate organ pathology, particularly in gills and liver tissues [33, 34]. Chronic exposure results in epithelial disintegration in the gills, impairing respiration and osmoregulation, and hepatocyte apoptosis in the liver, ultimately compromising metabolic and detoxification processes [35–37]. Moreover, heavy metals can interfere with endocrine pathways, impairing hormonal regulation essential for reproduction and resulting in poor oocyte development [38–40].

### Impact on gamete quality and reproductive function

The negative impact of heavy metals on reproductive health was further corroborated by gamete quality assessments. Oocytes from the control group exhibited the largest diameter, highest GVBD rates, and greatest viability, indicating optimal conditions. Conversely, oocytes from Kalisari displayed markedly reduced diameter, lower GVBD, and compromised survival, consistent with prior studies on the inhibitory effects of heavy metals on oocyte maturation and fertilization potential [41, 42]. Correlation analyses revealed a strong inverse relationship between metal concentrations and gamete quality metrics, indicating dose-dependent reproductive impairment. In addition, strong positive correlations between metal levels and histopathological lesion scores affirm the link between exposure intensity and tissue damage [34, 43].

### Ecological and public health implications

The pronounced histopathological damage in vital organs, alongside reduced reproductive capacity, highlights substantial ecological risks. Long-term contamination may Pb to reproductive failure and population decline, threatening aquatic biodiversity and ecosystem stability. Furthermore, the bioaccumulation of metals in edible fish tissues poses a direct health risk to humans, particularly in communities that rely on fish as a primary source of dietary protein. Chronic exposure through consumption can result in neurological and renal disorders, as emphasized by Ali *et al*. [8].

### Recommendations for environmental management

These findings underscore the urgent need for robust pollution control and environmental monitoring strategies. Effective mitigation should include stricter regulation of industrial discharge, improved wastewater treatment infrastructure, and routine ecological surveillance. Adopting a One Health approach perspective is essential to safeguard both aquatic ecosystems and public health [44].

## CONCLUSION

This study demonstrated that heavy metal contamination, particularly Cd and Fe, in the Brantas River Basin poses substantial ecotoxicological risks to freshwater fish, as evidenced by significant histopathological alterations and compromised reproductive health in female Nile tilapia (*O. niloticus*). The most severely affected site, Kalisari, exhibited the highest concentrations of Cd and Fe, correlating strongly with necrosis, inflammation, and hyperplasia in gill, liver, and ovarian tissues, as well as significantly reduced OD, GVBD rate, and survival.

These findings underscore the urgent need for environmental remediation and enhanced regulatory oversight to mitigate the discharge of heavy metals from industrial and residential sources. Monitoring Nile tilapia as a sentinel species offers a practical tool for bioindication and risk assessment in aquatic ecosystems. Moreover, the observed reproductive toxicity has implications for fishery sustainability and food safety, particularly in communities dependent on local fish stocks.

This study’s strength lies in its integrative approach, combining quantitative metal analysis, multi-organ histopathology, and detailed gamete quality metrics, to provide a comprehensive understanding of metal-induced toxicity under field conditions. The use of multiple ecologically relevant sampling sites and validated scoring systems further enhances the reliability of the findings.

However, the study has limitations. The sample size, while sufficient for detecting significant differences, may limit broader generalization. In addition, the cross-sectional design precludes causal inference over time. The exclusion of male fish and other life stages may overlook population-level effects.

Future research should include longitudinal studies to evaluate chronic and transgenerational effects of heavy metal exposure. Investigations into molecular biomarkers, hormonal profiles, and gene expression patterns will provide mechanistic insights. Moreover, comparative studies across species and seasons could better inform ecological risk assessments.

In summary, this study highlights the detrimental effects of heavy metal pollution on the integrity of fish organs and reproductive capacity. The findings necessitate immediate intervention through targeted pollution control, routine biomonitoring, and stakeholder collaboration within the One Health framework to preserve aquatic biodiversity and safeguard public health.

## DATA AVAILABILITY

The datasets generated and/or analyzed during the study are available from the corresponding author on reasonable request.

## AUTHORS’ CONTRIBUTIONS

HSAT, AA, and HA: Planned and designed the study. AA, GC, RB, SH, and PSA: Supervised the study, data analysis and interpretation, and revised the manuscript. HSAT and GR: Performed the field work and drafted the manuscript. All authors have read, reviewed, and approved the final manuscript.
